# The Effect of Triptolide Combined With Crocin on Arthritis in Mice: From Side Effect Attenuation to Therapy

**DOI:** 10.3389/fphar.2022.908227

**Published:** 2022-06-23

**Authors:** Min Yan, Yinyin Yan, Zhenqiang Zhang, Guoqiang Wang, Wenbo Shi, Mengyuan Jiang, Junwei Zhao, Xiangxiang Wu, Huahui Zeng

**Affiliations:** ^1^ Academy of Chinese Medicine Sciences, Henan University of Chinese Medicine, Zhengzhou, China; ^2^ School of Medicine, Henan University of Chinese Medicine, Zhengzhou, China; ^3^ Department of Clinical Laboratory, Core Unit of National Clinical Research Center for Laboratory Medicine, The First Affiliated Hospital of Zhengzhou University, Zhengzhou, China

**Keywords:** triptolide, crocin, compatibility, toxicity, arthritis

## Abstract

Clinical use of triptolide (TP) is restricted due to severe toxicity. This study assessed the protective effect of crocin (CR) as a natural antioxidant against TP-induced toxicity in bovine collagen type II-induced arthritis (CIA) in mice. The mice in the CIA model group showed macroscopic signs of severe arthritis. The anti-arthritis effects in the control, TP + CR, and TP groups were evaluated through assessment of foot volume, arthritis score, and proinflammatory cytokines, and collagen antibody assay. Crocin reduced TP-induced toxicity, as evidenced by evaluation of survival rate, body weight, visceral index, hepatic and renal functions, histopathologic analyses, and antioxidant enzyme activities. Transcriptome sequencing resulted in identification of 76 differentially expressed genes (DEGs) associated with hepatotoxicity between the TP and TP + CR groups. Of these, Three DEGs (Cyp1a2,Gsta4, and Gstp1) were validated using quantitative real-time PCR analysis. In conclusion, CR protected CIA mice from TP-induced toxicity through modulation of the cytochrome P450 and glutathione metabolism pathways.

## 1 Introduction

Triptolide (TP), the most studied bioactive chemical monomer of the Chinese herb Tripterygium wilfordii Hook. F., is a highly potent and effective anti-inflammatory, immunosuppressive, anti-rheumatoid, and anticancer agent (Tian et al., 2021; Tong et al., 2021; Zhang et al., 2021; Zhao et al., 2021).

Rheumatoid arthritis (RA) is a typical autoimmune disease often with symmetric facet joint disease, characterized by synovial hyperplasia, cartilage damages, and bone erosion. The collagen type II-induced arthritis (CIA) animal model (Trentham et al., 1977), is the most commonly studied model of RA (Zhao et al., 2022). In this model, antibodies against type II collagen play a crucial role for arthritis pathology (Yabe et al., 2021). The CIA model shares many pathological and histological similarities with RA, such as synovial hyperplasia, cartilage degradation and overproduction of inflammatory cytokines (Brand et al., 2007; Liang et al., 2018).

The Chinese herb Tripterygium wilfordii Hook. F. and its extracts have been used as an anti-rheumatic in China for many years (Qin, 2019). However, TP is highly toxic, suffers from poor aqueous solubility, and induces significant adverse effects, which limits its clinical use. Therefore, there is an urgent need to reduce TP-related toxicity without affecting therapeutic potency. Many strategies have been explored, including new dosage forms, structural modifications, and combination with other Chinese herbs (e.g., TP combined with chlorogenic acid or glycyrrhizic acid (GA)) (Tan et al., 2018; Wang et al., 2018; Zeng et al., 2020; Zhang et al., 2020; Yalikong et al., 2021).

Modern pharmacological studies of saffron and its main constituents have revealed a wide spectrum of biological activities (i.e., anti-inflammatory, antinociceptive, antioxidant, immunoregulatory effects, neurodegenerative diseases, cardiovascular diseases, anticancer, anti-arthritic effects and protection against natural and chemical toxins) (Attia et al., 2021; Xing et al., 2021). Crocin, mono, and diglycosyl esters of a polyene dicarboxylic acid are some of the main active components that are responsible for the pharmacological effects of saffron (Abdi et al., 2022; Salem et al., 2022; Xu et al., 2022).

Crocin (CR) is thought to protect against toxicity of viscera induced by some materials (El-Beshbishy et al., 2012; Razavi and Hosseinzadeh, 2015). In addition, pharmacokinetic studies have shown that crocin is not bioavailable after oral administration in blood circulation. Instead, it is rapidly transformed into crocetin in the gastrointestinal tract with high relative bioavailability (Zhang et al., 2017; Hosseini et al., 2018). However, there have been no studies to evaluate combination treatment with CR and TP as this treatment strategy may potentially reduce TP-related toxicity without impacting therapeutic efficacy. Therefore, this study focused on the mechanisms by which crocin mitigates TP-induced toxicity in a mouse bovine collagen type II-induced arthritis model.

## 2 Materials and Methods

### 2.1 Drugs, Reagents, and Animals

Triptolide (purity >98%) was purchased from Xi’an Haoxuan Biotechnology Co. Ltd. (Shanxi, China). Crocin (purity >98%) was obtained from TCI Chemical Industry (Shanghai, China). Glycyrrhizic acid (GA, purity 95%) was purchased from Cool Chemistry (Beijing, China).

Bovine type II collagen (2 mg/ml), Mouse Anti-Type II Collagen IgG Antibody ELISA Kits, complete Freund’s adjuvant (CFA, 4 mg/ml), and incomplete Freund’s adjuvant (IFA, 5 ml) were purchased from Condrex ((Norcross, GA, United States). BeyoRT™ III First Strand cDNA Synthesis Kit (Cat No. D7178M, Shanghai Biyuntian Biotechnology Co., Ltd., Shanghai, China), PowerUp™ SYBR™ Green Master Mix (Cat No. A25742, Thermo Fisher Scientific, United States), and kits for analysis of creatinine (CRE, Cat No. C011-2-1), blood urea nitrogen (BUN, Cat No. C013-1-1), alanine/aspartate transaminase (ALT/AST, Cat No. C009-2-1/C010-2-1), superoxide dismutase (SOD, Cat No. A001-1), malondialdehyde (MDA, Cat No. A003-1), catalase (CAT, Cat No. A007-2-1) and glutathione (GSH, Cat No. A006-2-1) were purchased from Nanjing Jiancheng Bioengineering Institute (Nanjing, China). Other chemicals and reagents used were of analytical grade.

Kunming (KM) mice (male, 7–8 weeks old, 20 ± 2 g) were purchased from Huaxing Laboratory Animal Farm (Zhengzhou, China) [License No: SCXK (Yu) 20190002]. Prior to experiments, all animals were acclimated to the experimental environment for 7 days and housed in a controlled environment (25 ± 1°C, humidity of 55 ± 5%) with a 12-h light/dark cycle. The mice were allowed free access to standard diet and water during the acclimation period. The experiments performed in this study were approved by the Animal Ethics Committee In Henan University of Chinese Medicine.

### 2.2 Collagen-Induced Arthritis Model and Grouping

**FIGURE 1:**
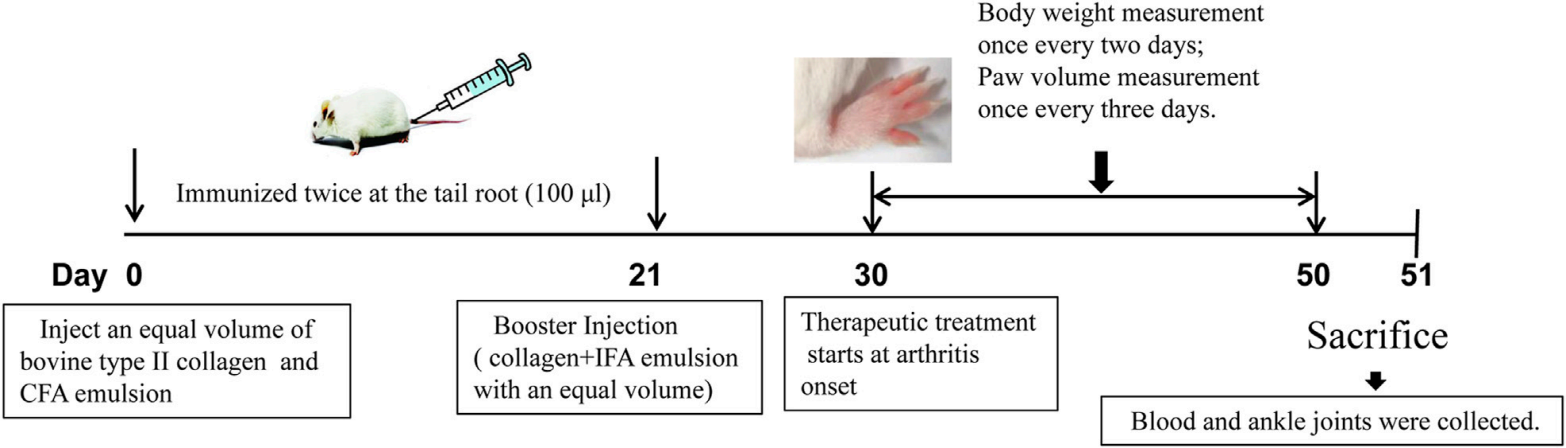
Schematic representation of CIA mouse model generation and treatment with TP or TP + CR. TP: triptolide; CR: crocin; CIA: collagen-induced arthritis.

The collagen-induced arthritis (CIA) model was established using bovine type II collagen, and the clinical severity of arthritis was scored as previously described [6]. Foot volume was measured using a volume-plethysmograph (PV-200, TECHMAN,Co. Ltd., Chengdu, China). The arthritis score (or deformation index) was used to describe the shape and joint swelling of the paws of CIA mice. Total scores for each mouse greater than eight were considered successful CIA models, and all mice in the control group had arthritis index scores of 0. A schematic for the animal modeling, mode of drug administration, and subsequent procedures is shown in Figure 1. After successful modeling, the mice were randomly divided into seven groups (n = 8) as follows:

A. normal saline (Control);
B. Collagen-induced arthritis model group (CIA);
C. 300 μg/kg TP treatment group (TP);
D. 300 μg/kg TP+50 mg/kg CR group (TP + LCR);
E. 300 μg/kg TP+100 mg/kg CR group (TP + MCR);
F. 300 μg/kg TP+200 mg/kg CR group (TP + HCR);
G. 300 μg/kg TP+50 mg/kg GA (TP + GA) as the positive drug control group (Yang et al., 2017).

A stock solution of TP was prepared in DMSO at 5 mg/ml, then diluted in saline to prepare the working solution. Doses and frequency of administration were determined during prior experiments. The animals were administered the indicated experimental treatments once every 2 days by oral gavage for 20 days (0.2 ml/10 g).

### 2.3 Drug Treatment Toxicity and Efficacy

Body weight, paw volume, and arthritis score were recorded during drug administration. Twenty-4 hours after the last drug treatment, the whole blood of each mouse was obtained via orbital collection. Serum was obtained by centrifugation (4°C, 3,000 rpm, 10 min). The serum levels of AST/ALT and BUN/CRE were quantified using specific commercial diagnostic kits. The levels of serum inflammatory cytokines (IL-1β, IL-6, and TNF-α) and mouse anti-type II collagen IgG were detected using ELISA kits according to the manufacturer’s instructions. The mice were sacrificed and the ankle joints were fixed in 10% formalin, decalcified, paraffin-embedded, and stained with hematoxylin and eosin (H&E).

### 2.4 *In Vivo* Drug Toxicity Study

To determine the mechanism by which CR protected against TP-induced toxicity, healthy mice were treated with TP + CR. The mice were randomly placed in the following seven groups (n = 10): A. Control (normal saline, NS); B. Low-dose TP (LTP, 100 μg/kg); C. Moderate-dose TP (MTP, 300 μg/kg); D. High-dose TP (HTP, 500 μg/kg); E. 100 μg/kg TP+100 mg/kg CR (LTP + CR); F. 300 μg/kg TP+100 mg/kg CR (MTP + CR); G. 500 μg/kg TP+100 mg/kg CR (HTP + CR). The mice were administrated the treatments daily via oral gavage for 7 days at a volume of 0.2 ml/10 g per dose. The mice were observed daily for 7 days to monitor body weight and mortality.

The mice were sacrificed and the organs (hearts, livers, spleens, lungs, kidneys, testes, stomach, and intestine) from each group were quickly harvested and washed with pre-cooled 0.9% saline solution to remove the blood. The visceral index was calculated as the ratio of visceral weight to body weight Using the following formula: Visceral index (%) = (viscera weight/body weight) × 100%. Hepatic and renal functional markers (AST/ALT and BUN/CRE) were measured as described in Section 2.3.

A portion of each sample was snap-frozen in liquid nitrogen and stored at −80°C for biochemical analysis. Portions of tissues were fixed in 10% formalin for hematoxylin and eosin (H&E) staining and visualized using a light microscope. Body weight, visceral index, histopathology, hematology, and biochemistry factors were investigated as part of the systemic toxicity evaluation.

### 2.5 Determination of Antioxidant Enzyme Activities

The livers were homogenized in cold saline and centrifuged at 12,000 rpm for 20 min at 4°C. The suspensions were used to assay SOD and CAT activities, and MDA and GSH levels using commercial detection kits according to the manufacturer’s instructions.

### 2.6 Transcriptome Sequencing (mRNA-Seq)

Liver tissues were selected from the Control, MTP, and MTP + CR groups for transcriptome studies. Library construction, mRNA-seq, and bioinformatic analysis were performed by Sinotech Genomics Co., Ltd. (Shanghai, China). The general workflow was as follows: RNA extraction and detection; mRNA enrichment and reverse transcription; cDNA purification, end repair, A-tailing, and sequencing adapters; and PCR enrichment and library construction. An Illumina NovaSeq 6000 (Illumina, United States) was used for RNA sequencing according to a previous study (Fu, Y. et al., 2020). The original data (raw reads) was trimmed to filter out unqualified sequences, then clean reads were mapped to the reference genome using Hisat2 (Hierarchical Indexing for Spliced Alignment of Transcripts, version 2.0.5). Gene abundance was expressed as fragments per kilobase of exon per million reads mapped (FPKM). Stringtie software was used to count the fragments within each gene, and the TMM algorithm was used for normalization. Differential expression analysis for mRNA was performed using R package edgeR. Differentially expressed RNAs with fold change values >1.5 and q values <0.05 were retained for further analysis, as these were considered as significantly modulated. The raw data are available under GEO: GSE202175.

### 2.7 Identification of Hepatotoxicity Targets

DisGeNet (http://www.disgenet.org/) and GeneCards (https://www.genecards.org/) were used to combine the relevant literature to generate a pool of hepatotoxicity targets with the keywords “hepatotoxicity or liver injury.” In the DisGeNet database, genes with EI ≥ 1 were selected. In the GeneCards database, genes with relevance scores ≥10 were selected. Finally, the combined targets were transferred from “*Homo sapiens*” to “*Mus musculus*” (https://string-db.org/).

### 2.8 Gene Ontology and Pathway Enrichment Analysis

Hepatotoxicity targets, differentially expressed mRNAs between the MTP group and the Control group, and differentially expressed mRNAs between the MTP + CR group and the MTP group were analyzed using a Venn diagram (http://bioinformatics.psb.ugent.be/webtools/Venn/). The intersecting genes in the Venn diagram were further investigated at the functional level. Gene Ontology (GO) analysis for biological processes, cellular components, and molecular function and KEGG (Kyoto Encyclopedia of Genes and Genomes) pathway analysis were performed using STRING database. The background species was defined as “*Mus musculus*."

### 2.9 Quantitative Real-Time PCR Analysis (qRT-PCR)

The relative levels of important differentially expressed genes (DEGs) identified in mRNA seq and KEGG analyses were selected for validation using qRT-PCR. Total RNA extracted from liver tissue using Trizol reagent (Invitrogen) for mRNA-seq was used for qRT-PCR. Total RNA was converted to cDNA according to the reverse transcription kit protocol. Then, PCR amplification was performed using SYBR green PCR master mix on an ABI 7500 FAST instrument. The primers for qRT-PCR are listed in Table 1. Glyceraldehyde-3-phosphate dehydrogenase (GAPDH) was used as the internal reference gene. The 2^-△△CT^ method was used to calculate the relative expression levels of the verified genes.

**TABLE 1:** Primer sequences for qRT-PCR.

Gene Name	Sequence (5'→3′)	Size (Bp)	NCBI GeneID
GSTP1	Forward ATG​CCA​CCA​TAC​ACC​ATT​GTC	161	14870
	Reverse GGG​AGC​TGC​CCA​TAC​AGA​C		
GSTA4	Forward TGA​TTG​CCG​TGG​CTC​CAT​TTA	135	14860
	Reverse CAA​CGA​GAA​AAG​CCT​CTC​CGT		
CYP1A2	Forward AGT​ACA​TCT​CCT​TAG​CCC​CAG	118	13077
	Reverse GGT​CCG​GGT​GGA​TTC​TTC​AG		
GAPDH	Forward AGG​TCG​GTG​TGA​ACG​GAT​TTG	123	14433
	Reverse TGT​AGA​CCA​TGT​AGT​TGA​GGT​CA		

### 2.10 Statistical Analysis

All data were processed using GraphPad Prism7 software and presented as the mean ± standard deviation ( $\bar{x}$ ±S). Statistical significance (*p* < 0.05, *p* < 0.01, or *p* < 0.001) was using Student’s *t*-test or one way ANOVA.

## 3 Results

### 3.1 Assessment of Drug Side Effects in Mice With Collagen-Induced Arthritis

**FIGURE 2:**
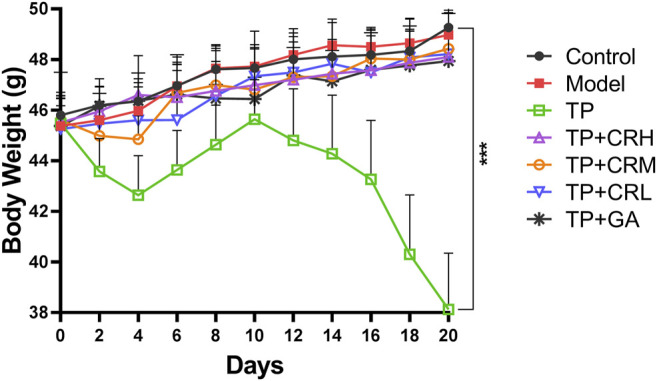
Body weight changes during 20 days of treatment. (n = 8) ***p < 0.001 vs Control.

**FIGURE 3:**
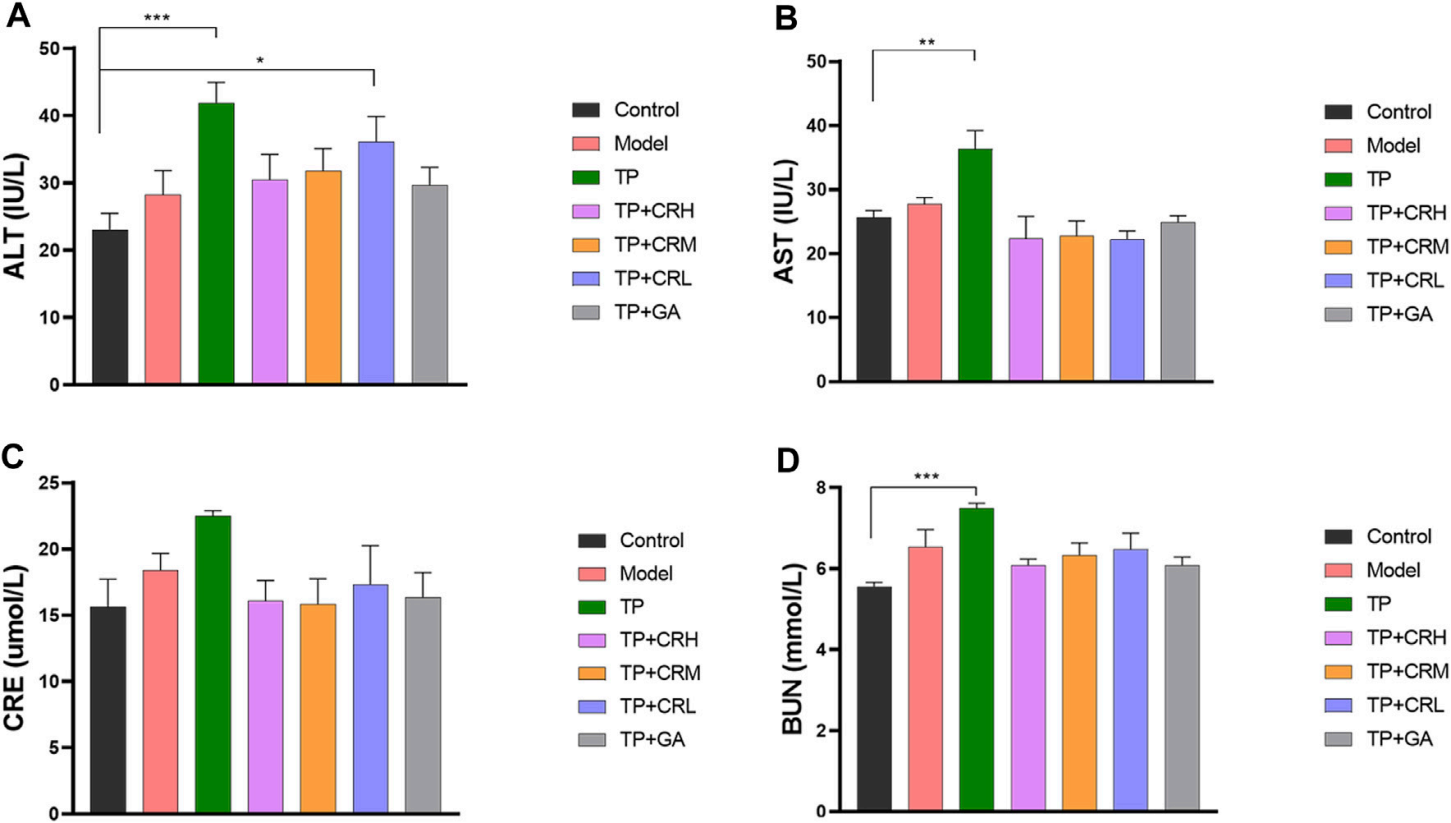
The levels of ALT **(A)**, AST **(B)**, CRE **(C)**, BUN **(D)** in the CIA mice liver were determined using respective kits, after 20 days of different treatment. (n = 8) **p < 0.01,***p < 0.001 vs. Control. ALT, alanine transaminase; AST, aspartate transaminase; CRE, creatinine; BUN, blood urea nitrogen; CIA, collagen-induced arthritis.

The side effects of the administered drugs on CIA mice were evaluated for 20 days after drug administrations. The parameters measured were changes in body weight, and hepatic and renal functions. The TP group showed obvious weight reduction compared to the other groups (*p* < 0.001), while other groups did not significantly differ from the control group (Figure 2). TP induced weight loss was significantly reversed by CR or GA combined treatment with TP. Compared with the control group, blood serum analysis demonstrated that the levels of AST, ALT, and BUN were significantly elevated in the TP group (*p* < 0.01). In contrast, co-administration of TP with CR or GA mitigated the changes observed in the TP group (Figure 3).

### 3.2 Evaluation of Therapeutic Effects in the CIA Model

**FIGURE 4:**
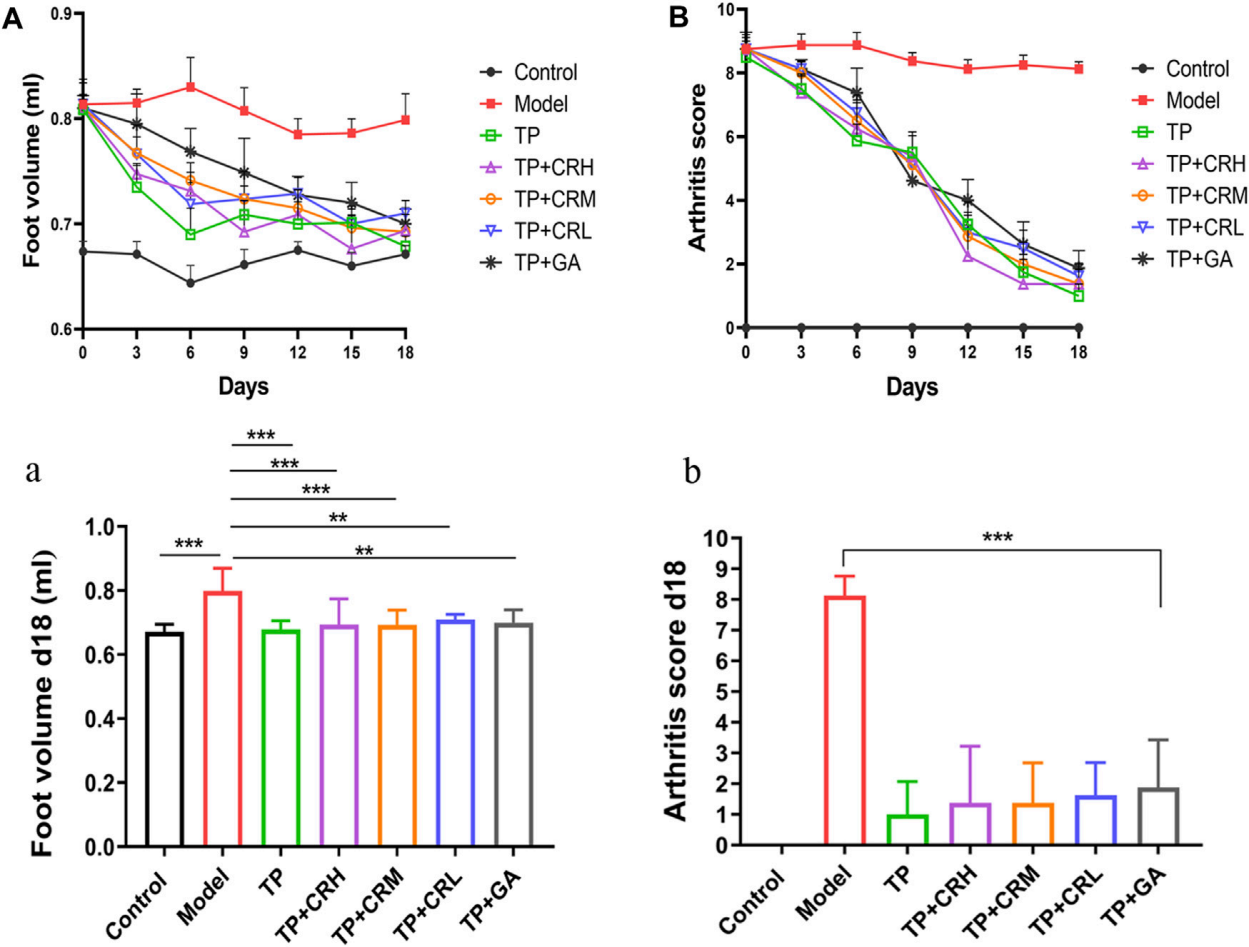
Changes in arthritic foot volume (**(A)**/a) and arthritis scores (**(B)**/b) in the CIA mice through the administration course (n = 8). **p < 0.01, ***p < 0.001 vs Control. CIA: collagen-induced arthritis.

Collagen-induced arthritis model mice were used to evaluate the therapeutic efficacy of TP + CR. Changes in foot volume and arthritis scores were evaluated every 3 days during drug treatment (Figure 4A,B). The foot volume and arthritis scores in the treatment groups were significantly lower than those in the model group at day 18 post-treatment (Figure 4 a,b).

**FIGURE 5:**
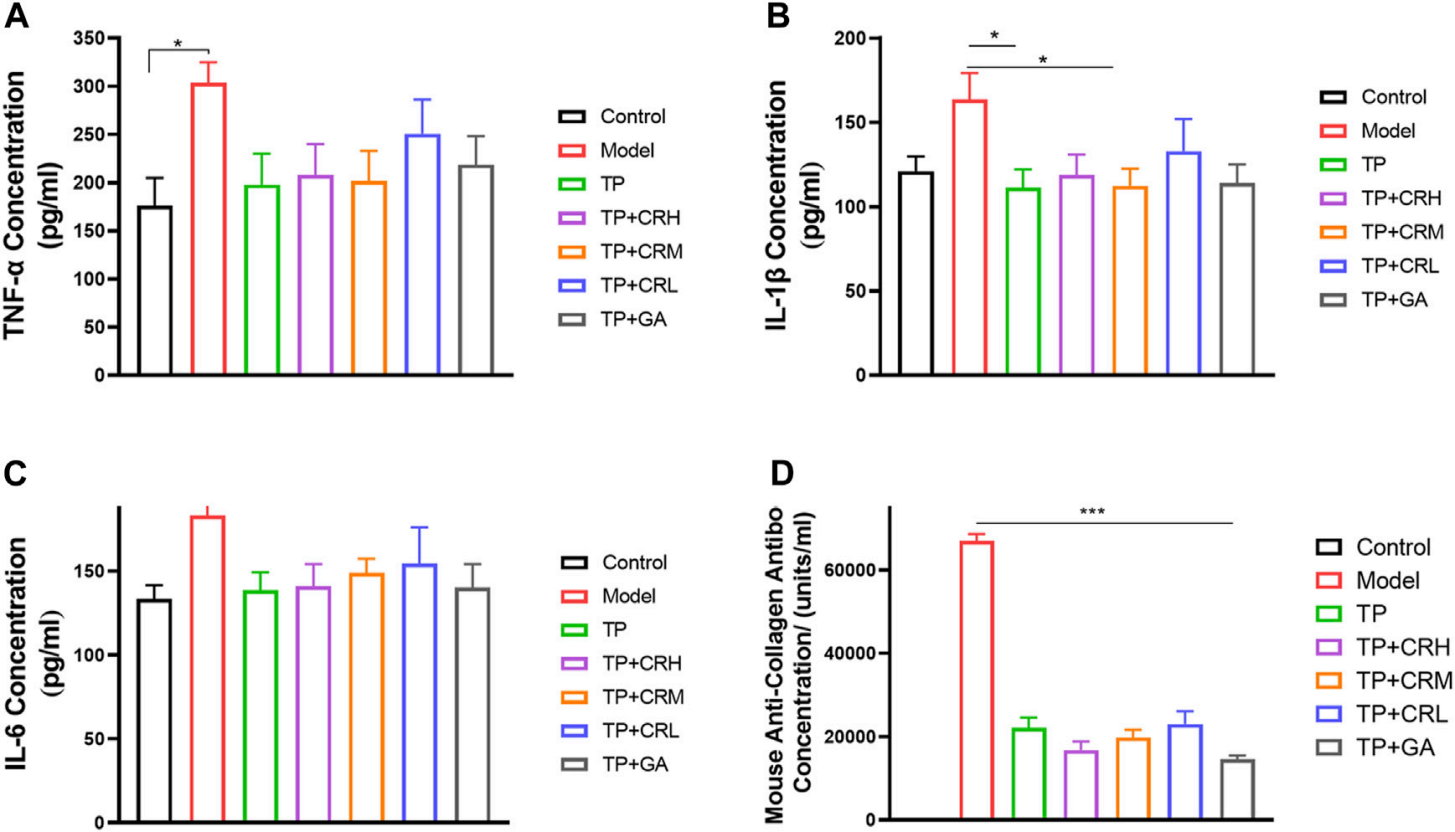
Quantitation of proinflammatory cytokines **(A)** TNF-α; **(B)** IL-1β; **(C)** IL-6 and anti-type II collagen antibody **(D)** in serum of each group using ELISA. (n = 6).

Levels of TNF-α, IL-1β, IL-6, and anti-type II collagen antibody in serum were quantitated using ELISA (Figure 5). The level of TNF-α in the serum of the model group was significantly higher than that in the control group (*p* < 0.05), and levels of IL-1β and IL-6 were increased, but the increases were not statistically significant. The three proinflammatory cytokines measured were reduced to different degrees in each treatment group compared with those in the model group. Anti-type II bovine collagen antibody was not detected in the control group, and was significantly increased in the model group (*p* < 0.001). The levels of collagen antibody were lower in each administration group compared with those in the model group (*p* < 0.001).

**FIGURE 6:**
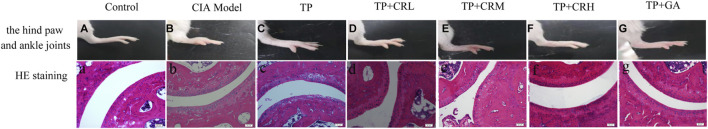
Photos of right hind paws **(A–G)** and histopathological analysis of ankle joints stained with H&E **(a–g)**.

Mice in the CIA group showed macroscopic signs of severe arthritis such as deformity, swelling, and redness in the hind paw and ankle joints (Figure 6B). Foot swelling was significantly reduced in each treatment group compared with that in the Model group (Figure 6C–G). As shown in Figures 6a,b, the histological tissue sections showed that the surfaces of the ankle joints in the control group had normal morphology and smooth articular cartilage. In contrast, structural damage and edema of the ankle joint, synovial hyperplasia, and joint cavitation were observed in the CIA model group. The treatment groups showed differing degrees of improvement compared with the Model group (Figure 6C–G). In particular, the TP + CRH and TP + GA groups showed normal morphology similar to that in the Control group.

### 3.3 Evaluation of Toxicity of TP + CR in Normal Kunming Mice

**FIGURE 7:**
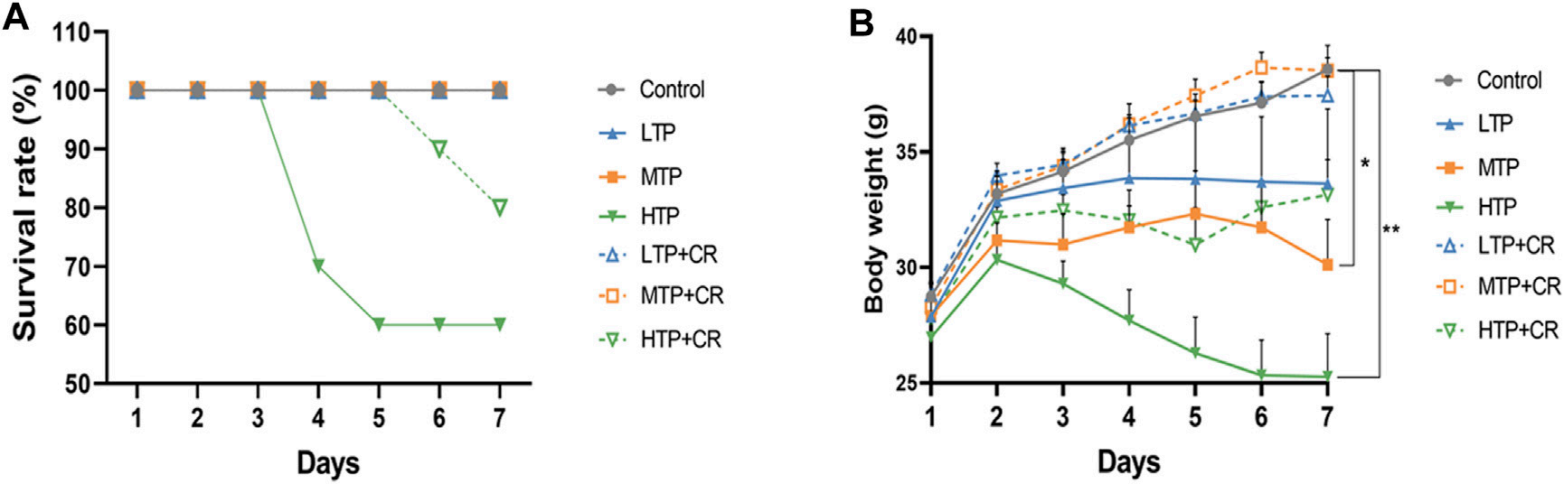
The survival rates **(A)** (n = 10) and body weight changes **(B)** (n = 6) of different groups over 7 days *p < 0.05, **p < 0.01 vs Control.

Survival rates and body weights were evaluated 7 days after treatment administration (Figure 7). The HTP and HTP + CR groups were the only groups in which mice died. The survival rate in the HTP + CR group was significantly higher than that in the HTP group. Body weight was lower on the seventh day in all but the LTP + CR and MTP + CR groups compared with that in the control group. Treatment with MTP or HTP induced significant weight loss. Furthermore, the MTP and HTP groups showed significantly lower organ coefficients (*p* < 0.05), while the other groups did not significantly differ from the control group (Table 2). The weight indices of multiple organs appeared normal following co-administration of TP and CR. In addition, biochemical analysis was performed to evaluate drug-induced hepatic and renal damage. As shown in Table 3, the serum ALT/AST and BUN/CRE levels were significantly elevated following TP administration compared with those in the control and the co-administration groups. Co-administration of TP and CR group did not induce changes in serum ALT/AST or BUN/CRE compared to the control group. These results showed that administration of CR significantly mitigated TP-induced changes in hepatic and renal functions.

**TABLE 2:** Comparison of organ coefficients in each group ( $\bar{x}$ ± S, n = 6).

Groups	Heart	Liver	Spleen	Lung	Kidney	Testis
Control	0.569 ± 0.019	5.665 ± 0.410	0.514 ± 0.090	0.729 ± 0.074	1.437 ± 0.117	0.635 ± 0.049
LTP	0.544 ± 0.022	5.301 ± 0.342	0.507 ± 0.041	0.682 ± 0.052	1.405 ± 0.087	0.604 ± 0.123
MTP	0.530 ± 0.023**	5.086 ± 0.345*	0.405 ± 0.064*	0.641 ± 0.055*	1.239 ± 0.158*	0.561 ± 0.038*
HTP	0.476 ± 0.036**	4.936 ± 0.413*	0.388 ± 0.092*	0.591 ± 0.052**	1.165 ± 0.202*	0.482 ± 0.112*
LTP + CR	0.564 ± 0.030	5.530 ± 0.455	0.509 ± 0.114	0.714 ± 0.063	1.424 ± 0.075	0.629 ± 0.037
MTP + CR	0.563 ± 0.029^#^	5.527 ± 0.233^#^	0.491 ± 0.026^#^	0.725 ± 0.066^#^	1.428 ± 0.093^#^	0.624 ± 0.050^#^
HTP + CR	0.548 ± 0.051^#^	5.499 ± 0.306^#^	0.499 ± 0.024^#^	0.704 ± 0.092^#^	1.400 ± 0.048^#^	0.621 ± 0.101^#^

Notes: *p < 0.05, **p < 0.01 any group vs Control; ^#^ *p* < 0.05, XTP + CR, vs XTP (X represent L, M, or H).

**TABLE 3:** Comparison of hepatic and renal functions among the groups of mice ( $\bar{x}$ ± S, n = 6).

Groups	AST (IU/L)	ALT (IU/L)	BUN(mmol/L)	CRE(μmol/L)
Control	23.373 ± 2.829	30.512 ± 9.592	4.634 ± 0.291	14.037 ± 1.952
LTP	34.526 ± 6.497**	43.86 ± 7.142*	4.953 ± 0.221	16.931 ± 2.284
MTP	41.998 ± 4.595**	45.382 ± 10.674*	5.609 ± 0.188**	21.782 ± 1.902*
HTP	50.125 ± 7.816**	49.224 ± 15.304*	7.948 ± 1.171**	22.802 ± 2.866*
LTP + CR	24.32 ± 8.476^#^	26.774 ± 12.049^#^	4.781 ± 0.195	15.259 ± 1.229
MTP + CR	25.064 ± 6.311^##^	30.371 ± 4.409^#^	4.916 ± 0.480^##^	15.625 ± 0.914^##^
HTP + CR	25.428 ± 5.766^##^	34.528 ± 3.703^#^	5.017 ± 0.485^##^	17.533 ± 3.445^#^

Notes: *p < 0.05, **p < 0.01 any group vs Control; ^#^ *p* < 0.05, ^##^ *p* < 0.01, XTP + CR, vs XTP (X represent L, M, or H).

### 3.4 Crocin Alleviates Damage of Histological Structure Induced by TP Toxicity

**FIGURE 8:**
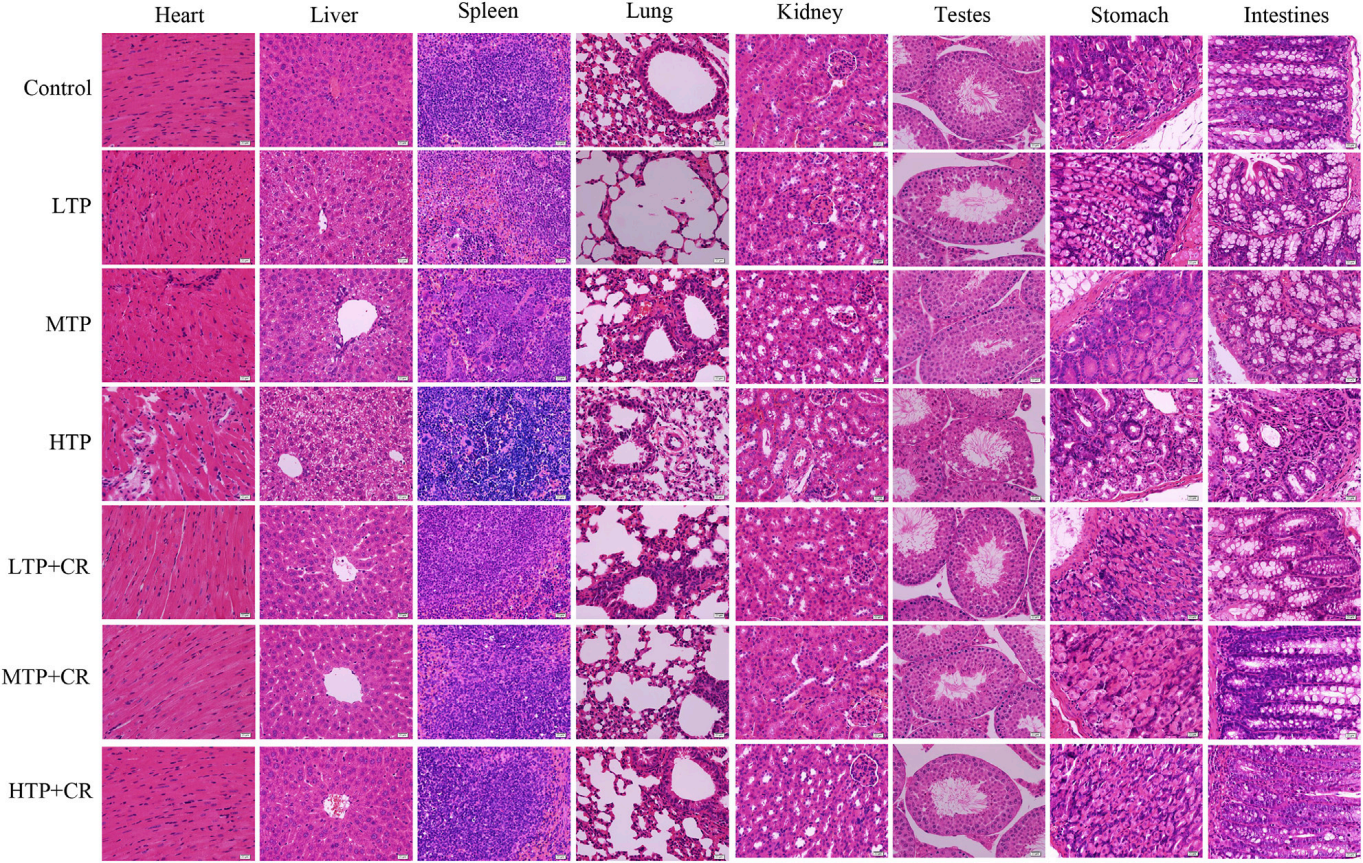
Histopathological observations of H&E staining on the main visceral organs of the mice. Images were obtained at 400X magnification (scale bar = 20 μm).

We performed histopathological analyses of H&E-stained tissue sections from hearts, livers, spleens, lungs, kidneys, testes, stomach, and intestines. As shown in Figure 8 and Table 4, tissue injury was significantly reduced in the TP + CR group.

**TABLE 4:** Morphological and pathological manifestations of mice in each group.

	Control	TP Groups	TP + CR Groups
Heart	The myocardial fibers were orderly and tightly arranged	LTP and MTP groups: the transverse striations were ambiguous and there was interstitial congestion	Myocardial damage was significantly ameliorated and histological morphology tended to be normal
		HTP group: cardiomyocytes had been dissolved, interstitial fibers showed hyperplasia, and inflammatory cell infiltration occurred	
Liver	Normal cell morphology, clear hepatic lobule structure, and neatly arranged hepatocytes with no inflammatory	LTP group: few inflammatory cells were observed	Clear structure with no edema, and small fat vacuoles were observed in part of the hepatocyte cytoplasm. Hepatocellular injury was significantly improved
	cell infiltration	MTP group: hepatocyte edema and punctate necrosis	
		Disordered hepatic cell cord arrangement, and compressed sinusoids	
		HTP group: hepatocytes exhibited diffuse hydropic or fatty degeneration	
Spleen	Splenic corpuscle was circular and the structure was clear	LTP group: Decreased lymphocyte density, increased red pulp macrophages	White pulp injury was significantly reduced and no abnormal changes were observed
	MTP group: Significant increase in the number of multinucleated macrophages and erythrocytes		
	HTP group: white pulp structures were disordered and numerous apoptotic bodies were present		
Lung	The alveolar structure in H&E staining was normal, the lung tissue was well structured, and there was no inflammatory cell infiltration	MTP and HTP group: alveolar and alveolar interstitial inflammatory cell infiltration and edema	Normal alveoli and few inflammatory cells
Kidney	Tubular epithelial cells and glomerular structures were clear and intact	With increased TP drug dosage, the proximal convoluted tubules were edematous and glomerular capillaries were markedly dilated. Infiltration of inflammatory cells was observed	Normal histological morphology. Slight hyperplasia of fibrous tissue was observed in the interstitium
Testes	Clear structure of seminiferous tubules and orderly arrangement of spermatogenic cells. Mature sperm were observed	Compared to that in the healthy testes, the number of spermatogenic cells in the seminiferous tubules was reduced, and they were disorganized	Compared with the model group, the number of spermatogenic cells was increased and the cells were arranged in an orderly fashion
Stomach	The control group showed a normal structure and no histopathological changes	HTP group: tissue structure was disordered, mucosal epithelial cells had shed, and inflammatory cell infiltration was observed	CR treatment significantly ameliorated gastric mucosa damage; regular glandular structure was observed, and reduced inflammatory cell infiltration
Intestine	The mucosal epithelial cells were arranged neatly	HTP group: The epithelial cells had shed and inflammatory cells had infiltrated the lamina propria	The tissues were clear and intact

### 3.5 Determination of Antioxidant Enzyme Activities

**FIGURE 9:**
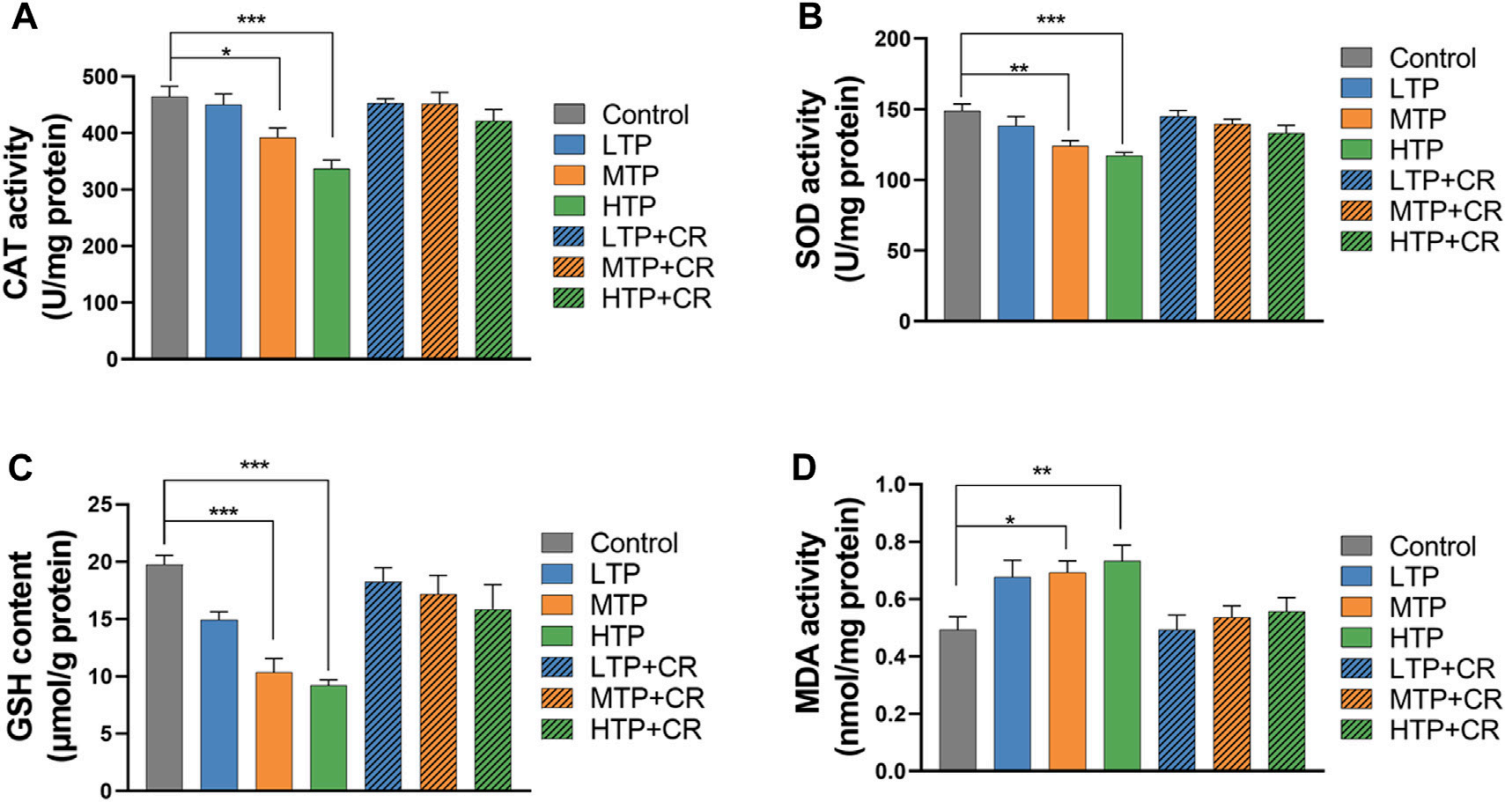
Levels of CAT **(A)**, SOD **(B)**, GSH **(C)**, and MDA **(D)** in liver homogenates of mice (n = 6). *p < 0.05, **p < 0.01, ***p < 0.001 vs Control.

As shown in Figure 9, the activities of hepatic CAT and SOD, and the levels of GSH and MDA, were measured in each group. Hepatic SOD and CAT activities, and GSH content, decreased in a TP dose-dependent manner (*p* < 0.01). There were no significant differences between the TP + CR group and the control groups. Moreover, MDA content was higher in the MTP and HTP groups than in the control group (*p <* 0.05). However, there was no significant difference in MDA content in the liver between the TP + CR group and the control group.

### 3.6 Differentially Expressed mRNAs and KEGG Analysis

**FIGURE 10:**
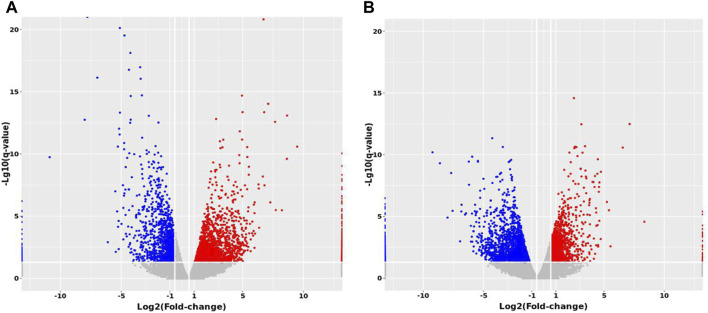
Volcano maps of differentially expressed mRNAs between groups. **(A)** MTP vs. Control **(B)** MTP + CR vs. MTP.

Expression profiling studies were performed on the RNAs from four independent liver tissue samples in each group. The volcano plot of differentially expressed mRNAs among three groups is shown in Figure 10.

**FIGURE 11:**
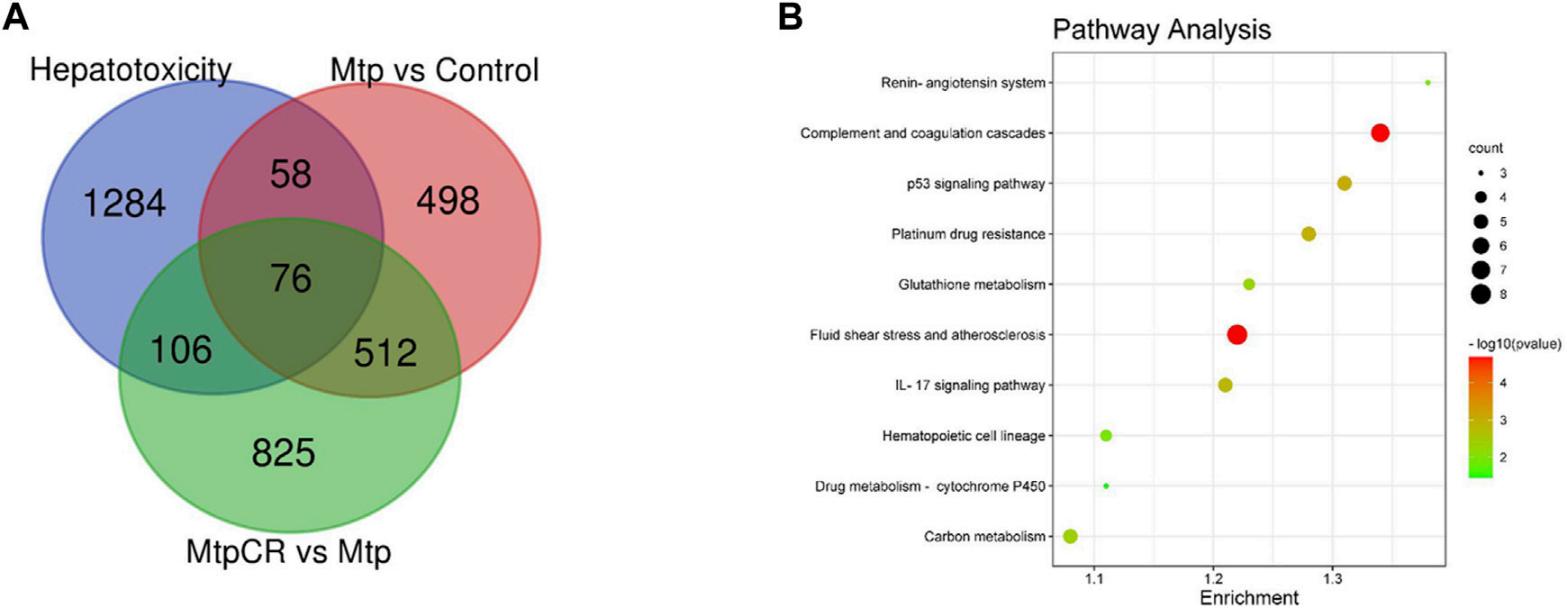
Venn diagram showing the number of differently expressed mRNAs related to hepatotoxicity **(A)** and pathway enrichment analysis **(B)**.

Compared with the Control group, 1,148 mRNAs were identified to be differently expressed in the MTP groups. Furthermore, 1,526 mRNAs were differentially expressed in the MTP + CR group compared with the MTP group (Figure 11A). There were 76 differentially expressed mRNAs identified in the MTP group compared with Control group as well as in the MTP + CR group compared with MTP group, which were also associated with hepatotoxicity. Kyoto Encyclopedia of Genes and Genomes analysis showed that the differentially expressed mRNAs related to hepatotoxicity were mainly enriched in the complement and coagulation cascades, p53 signaling pathway, glutathione metabolism, IL-17 signaling pathway, and drug metabolism-cytochrome P450 (Figure 11B).

### 3.7 Validation of RNA-Seq Results Using qRT-PCR

**FIGURE 12:**
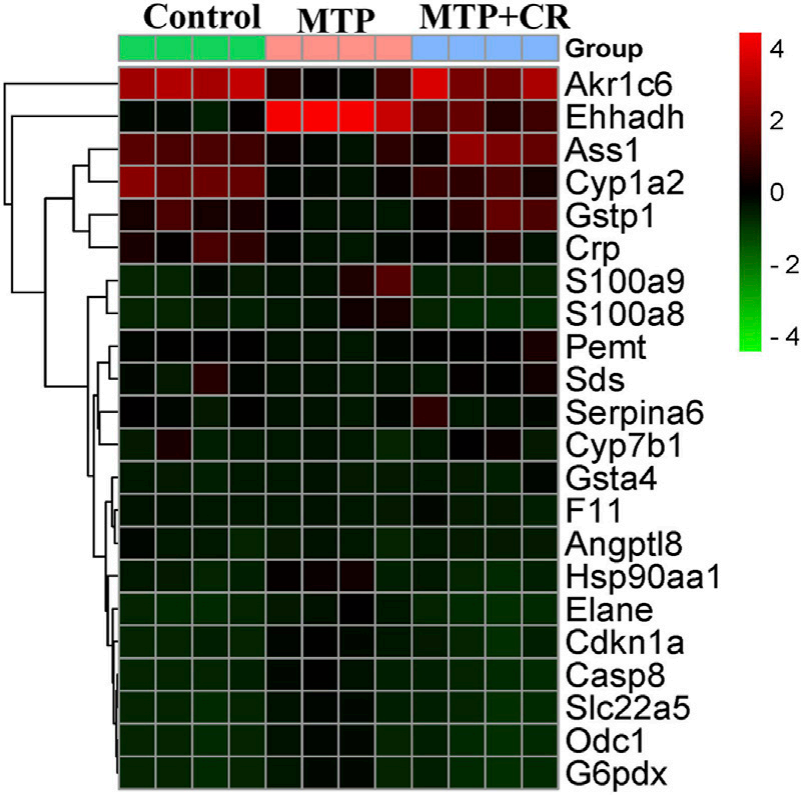
The heat maps of the Top 23 differentially-expressed mRNAs among three groups.

**FIGURE 13:**
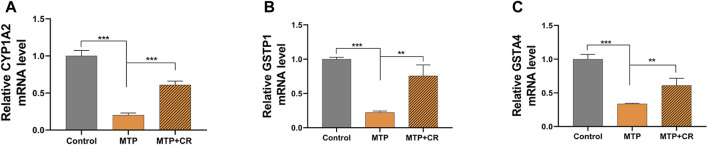
Confirmation of mRNA expression by qRT-PCR **(A)** the CYP1A2 mRNA level; **(B)** the GSTP1 mRNA level; **(C)** the GSTA4 mRNA level. **p < 0.01,***p < 0.001 vs MTP.

Expression of mRNA was restricted to FPKM > 20, which resulted in selection of 23 DEGs. Analysis of the expression trends of the top 23 DEGs (Figure 12) resulted in selection of Cyp1a2, Gstp1, and Gsta4, which were related to drug metabolism-cytochrome P450 and glutathione metabolism, for further qRT-PCR verification. As shown in Figure 13, Cyp1a2, Gsta4, and Gstp1 expression levels were decreased in the MTP group, and these decreases were mitigated in the MTP + CR group.

## 4 Discussion

The therapeutic window for TP is very narrow, resulting in sometimes overlapping therapeutic and toxic ranges. Therefore, identification of methods to attenuate toxicity without impacting efficacy is of great importance.

Previous studies showed that crocin has a considerable anti-inflammatory and antioxidant potency (Li et al., 2018; Korani et al., 2019; Yaribeygi et al., 2021). In addition, combined treatment with GA and TP increase efficacy while decreasing toxicity in RA treatment (Tai et al., 2014). Therefore, in this study, we explored the ability of the novel treatment combination of TP + CR to treat arthritis with reduced TP-induced toxicity using GA combined with TP as the positive control. As a result, the anti-arthritis effects in the TP + CR group were equivalent to those in the TP and TP + GA groups, as determined by evaluation of foot volume and arthritis score, proinflammatory cytokine levels, and collagen antibody assay. Animal toxicity experiments showed that CR reduced TP-induced multi-organ damage (Figure 8 and Table 4), and reduced mortality.

Hepatotoxicity is the most significant TP-induced side effect and the mechanism is complex, which is believed to occur via oxidative stress, abnormal liver drug enzyme activity, and immune-mediated injury (Tian et al., 2019). Crocin has been shown to act on multiple pharmacological targets, such as antioxidant, anti-inflammatory, and immunoregulatory systems that may be involved in TP-induced toxicity (Attia et al., 2021).

Previous animal studies have shown that crocin is a protective against doxorubicin-induced nephrotoxicity and cyclophosphamide-induced hepatotoxicity through modulation of antioxidant enzymes and inflammatory mediators (Jnaneshwari et al., 2013; Hussain et al., 2021). Combined treatment with vitamin C and TP could combat oxidative stress by regulating the levels of SOD, GSH, CAT and MD (Xu et al., 2019), which agrees with the known mechanisms for TP-induced hepatotoxicity (Cao et al., 2022). TP may lead to emergency oxidative damage of liver cells by reducing thioredoxin activity (Shen et al., 2019). In this study, we evaluated changes in oxidative stress markers in the liver, the primary organ responsible for drug metabolism. The results showed that co-administration of TP + CR reversed TP-induced changes in SOD and CAT activities, and GSH and MDA levels, in a dose-dependent manner.

The metabolism of TP and other drugs depends on enzymes in liver microsomes, which involve phase I and phase II metabolism. Cytochrome P450s, representative enzymes of Phase I, are responsible for the hydroxylation of triptolide *in vitro* and CYP3A4 enzyme mediated metabolic elimination is an important detoxification pathway for TP (Xiao et al., 2020). Glutathione-S-transferases are a superfamily of phase-II metabolic enzymes that protect against oxidative stress (Singh and Reindl, 2021). Transcriptome sequencing and KEGG analysis in this study showed that differentially expressed mRNAs related to hepatotoxicity (76 mRNAs) were mainly enriched in glutathione metabolism, IL-17 signaling pathway, and drug metabolism-cytochrome P450. We selected the most significantly differentially expressed genes, Cyp1a2, Gstp1, and Gsta4, for confirmation by qRT-PCR. Our results showed that co-administration of TP + CR reversed TP-induced decreased in Cyp1a2, Gsta4, and Gstp1 expression. Cyp1a2 is an important phase-I metabolic enzyme in the cytochrome P450 family of enzymes. Gsta4 and Gstp1 can catalyze the binding of glutathione to TP metabolites *in vivo*, thus reducing the toxicity of TP. Therefore, our results indicated that phase I and phase II metabolic enzymes both participate in critical detoxification processes in TP-induced liver injury, and crocin could reduce TP-induced liver injury by down-regulating gene expression of these enzymes. In addition, IL-17 mediated the immune response of TP and played an essential role in the liver injury pathology (Wei et al., 2017). Our results also revealed that crocin might be involved in IL-17 mediated immune regulation. However, further research is still required.

In conclusion, our results demonstrated that co-administration of TP and CR could protect CIA mice from TP-induced multi-organ damage without reducing the therapeutic efficacy of TP. The mechanisms by which CR protected against TP-induced toxicity may have been related to the drug metabolism-cytochrome P450 and glutathione metabolism pathways. Our study demonstrated that CR could be used to attenuate TP toxicity without impacting therapeutic efficacy. Future studies should clarify the specific mechanisms by which CR protects against TP-induced toxicity, and ongoing studies are exploring new dosage forms and administration strategies for CR and TP.

## Data Availability

The datasets presented in this study can be found in online repositories. The names of the repository/repositories and accession number(s) can be found below: https://www.ncbi.nlm.nih.gov/geo/, GSE202175

## References

[B1] Abdi H. Aganj Z. Hosseinzadeh H. Mosaffa F. (2022). Crocin Restores the Balance of Th1/Th2 Immune Cell Response in ConA-Treated Human Lymphocytes. Pharmacol. Rep. 74, 513–522. *[Preprint]* . 10.1007/s43440-022-00362-3 35294736

[B2] Attia A. A. Ramdan H. S. Al-Eisa R. A. Adle Fadle B. O. A. El-Shenawy N. S. (2021). Effect of Saffron Extract on the Hepatotoxicity Induced by Copper Nanoparticles in Male Mice. Molecules 26 (10), 3045. 10.3390/molecules26103045 34065267 PMC8161208

[B3] Brand D. D. Latham K. A. Rosloniec E. F. (2007). Collagen-induced Arthritis. Nat. Protoc. 2 (5), 1269–1275. 10.1038/nprot.2007.173 17546023

[B4] Cao Z. Liu B. Li L. Lu P. Yan L. Lu C. (2022). Detoxification Strategies of Triptolide Based on Drug Combinations and Targeted Delivery Methods. Toxicology 469, 153134. 10.1016/j.tox.2022.153134 35202762

[B5] El-Beshbishy H. A. Hassan M. H. Aly H. A. Doghish A. S. Alghaithy A. A. (2012). Crocin "saffron" Protects against Beryllium Chloride Toxicity in Rats through Diminution of Oxidative Stress and Enhancing Gene Expression of Antioxidant Enzymes. Ecotoxicol. Environ. Saf. 83, 47–54. 10.1016/j.ecoenv.2012.06.003 22766413

[B6] Hosseini A. Razavi B. M. Hosseinzadeh H. (2018). Pharmacokinetic Properties of Saffron and its Active Components. Eur. J. Drug. Metab. Pharmacokinet. 43 (4), 383–390. 10.1007/s13318-017-0449-3 29134501

[B7] Hussain M. A. Abogresha N. M. AbdelKader G. Hassan R. Abdelaziz E. Z. Greish S. M. (2021). Antioxidant and Anti-inflammatory Effects of Crocin Ameliorate Doxorubicin-Induced Nephrotoxicity in Rats. Oxid. Med. Cell. Longev. 2021, 8841726. 10.1155/2021/8841726 33628387 PMC7899759

[B8] Jnaneshwari S. Hemshekhar M. Santhosh M. S. Sunitha K. Thushara R. Thirunavukkarasu C. (2013). Crocin, a Dietary Colorant, Mitigates Cyclophosphamide-Induced Organ Toxicity by Modulating Antioxidant Status and Inflammatory Cytokines. J. Pharm. Pharmacol. 65 (4), 604–614. 10.1111/jphp.12016 23488790

[B9] Korani S. Korani M. Sathyapalan T. Sahebkar A. (2019). Therapeutic Effects of Crocin in Autoimmune Diseases: A Review. BioFactors 45 (6), 835–843. 10.1002/biof.1557 31430413

[B10] Li L. Zhang H. Jin S. Liu C. (2018). Effects of Crocin on Inflammatory Activities in Human Fibroblast-like Synoviocytes and Collagen-Induced Arthritis in Mice. Immunol. Res. 66 (3), 406–413. 10.1007/s12026-018-8999-2 29777367

[B11] Liang H. Peng B. Dong C. Liu L. Mao J. Wei S. (2018). Cationic Nanoparticle as an Inhibitor of Cell-free DNA-Induced Inflammation. Nat. Commun. 9 (1), 4291. 10.1038/s41467-018-06603-5 30327464 PMC6191420

[B12] Qin W. Z. (2019). “Chapter 2 Rheumatoid Arthritis,” in The Study on Tripterygium Wilfordii. Editor Tu S (BeiJing, China: science press), 378–387.

[B13] Razavi B. M. Hosseinzadeh H. (2015). Saffron as an Antidote or a Protective Agent against Natural or Chemical Toxicities. Daru 23 (1), 31. 10.1186/s40199-015-0112-y 25928729 PMC4418072

[B14] Salem M. Shaheen M. Tabbara A. Borjac J. (2022). Saffron Extract and Crocin Exert Anti-inflammatory and Anti-oxidative Effects in a Repetitive Mild Traumatic Brain Injury Mouse Model. Sci. Rep. 12 (1), 5004. 10.1038/s41598-022-09109-9 35322143 PMC8943204

[B15] Shen F. Xiong Z. Kong J. Wang L. Cheng Y. Jin J. (2019). Triptolide Impairs Thioredoxin System by Suppressing Notch1-Mediated PTEN/Akt/Txnip Signaling in Hepatocytes. Toxicol. Lett. 300, 105–115. 10.1016/j.toxlet.2018.10.024 30394310

[B16] Singh R. R. Reindl K. M. (2021). Glutathione S-Transferases in Cancer. Antioxidants (Basel) 10 (5), 701. 10.3390/antiox10050701 33946704 PMC8146591

[B17] Tai T. Huang X. Su Y. Ji J. Su Y. Jiang Z. (2014). Glycyrrhizin Accelerates the Metabolism of Triptolide through Induction of CYP3A in Rats. J. Ethnopharmacol. 152 (2), 358–363. 10.1016/j.jep.2014.01.026 24486211

[B18] Tan Q. Y. Hu Q. Zhu S. N. Jia L. L. Xiao J. Su H. Z. (2018). Licorice Root Extract and Magnesium Isoglycyrrhizinate Protect against Triptolide-Induced Hepatotoxicity via Up-Regulation of the Nrf2 Pathway. Drug. Deliv. 25 (1), 1213–1223. 10.1080/10717544.2018.1472676 29791258 PMC6058668

[B19] Tian Y. Li P. Xiao Z. Zhou J. Xue X. Jiang N. (2021). Triptolide Inhibits Epithelial-Mesenchymal Transition Phenotype through the p70S6k/GSK3/β-Catenin Signaling Pathway in Taxol-Resistant Human Lung Adenocarcinoma. Transl. Lung. Cancer. Res. 10 (2), 1007–1019. 10.21037/tlcr-21-145 33718039 PMC7947389

[B20] Tian Y. G. Su X. H. Liu L. L. Kong X. Y. Lin N. (2019). Overview of Hepatotoxicity Studies on Tripterygium Wilfordii in Recent 20 Years. Zhongguo. Zhong. Yao. Za. Zhi. 44 (16), 3399–3405. 10.19540/j.cnki.cjcmm.20190527.408 31602901

[B21] Tong L. Zhao Q. Datan E. Lin G. Q. Minn I. Pomper M. G. (2021). Triptolide: Reflections on Two Decades of Research and Prospects for the Future. Nat. Prod. Rep. 38 (4), 843–860. 10.1039/d0np00054j 33146205

[B22] Trentham D. E. Townes A. S. Kang A. H. (1977). Autoimmunity to Type II Collagen an Experimental Model of Arthritis. J. Exp. Med. 146 (3), 857–868. 10.1084/jem.146.3.857 894190 PMC2180804

[B23] Wang J. M. Chen R. X. Zhang L. L. Ding N. N. Liu C. Cui Y. (2018). *In Vivo* protective Effects of Chlorogenic Acid against Triptolide-Induced Hepatotoxicity and its Mechanism. Pharm. Biol. 56, 626–631. 10.1080/13880209.2018.1527370 31070533 PMC6300082

[B24] Wei C. B. Tao K. Jiang R. Zhou L. D. Zhang Q. H. Yuan C. S. (2017). Quercetin Protects Mouse Liver against Triptolide-Induced Hepatic Injury by Restoring Th17/Treg Balance through Tim-3 and TLR4-MyD88-NF-Κb Pathway. Int. Immunopharmacol. 53, 73–82. 10.1016/j.intimp.2017.09.026 29040945

[B25] Xiao X. Zhang T. Huang J. Zhao Q. Li F. (2020). Effect of CYP3A4 on Liver Injury Induced by Triptolide. Biomed. Chromatogr. 34, e4864. 10.1002/bmc.4864 32330997

[B26] Xing B. Li S. Yang J. Lin D. Feng Y. Lu J. (2021). Phytochemistry, Pharmacology, and Potential Clinical Applications of Saffron: A Review. J. Ethnopharmacol. 281, 114555. 10.1016/j.jep.2021.114555 34438035

[B27] Xu P. Li Y. Yu Z. Yang L. Shang R. Yan Z. (2019). Protective Effect of Vitamin C on Triptolide-Induced Acute Hepatotoxicity in Mice through Mitigation of Oxidative Stress. An. Acad. Bras. Cienc. 91, e20181257. 10.1590/0001-3765201920181257 31241707

[B28] Xu Z. Lin S. Tong Z. Chen S. Cao Y. Li Q. (2022). Crocetin Ameliorates Non-alcoholic Fatty Liver Disease by Modulating Mitochondrial Dysfunction in L02 Cells and Zebrafish Model. J. Ethnopharmacol. 285, 114873. 10.1016/j.jep.2021.114873 34848360

[B29] Yabe R. Chung S. H. Murayama M. A. Kubo S. Shimizu K. Akahori Y. (2021). TARM1 Contributes to Development of Arthritis by Activating Dendritic Cells through Recognition of Collagens. Nat. Commun. 12, 94. 10.1038/s41467-020-20307-9 33397982 PMC7782728

[B30] Yalikong A. Li X. Q. Zhou P. H. Qi Z. P. Li B. Cai S. L. (2021). A Triptolide Loaded HER2-Targeted Nano-Drug Delivery System Significantly Suppressed the Proliferation of HER2-Positive and BRAF Mutant Colon Cancer. Int. J. Nanomedicine. 16, 2323–2335. 10.2147/IJN.S287732 33776436 PMC7989962

[B31] Yang G. Wang L. Yu X. Huang Y. Qu C. Zhang Z. (2017). Protective Effect of 18β-Glycyrrhetinic Acid against Triptolide-Induced Hepatotoxicity in Rats. Evidence-Based Complementary Altern. Med. 2017, 3470320. 10.1155/2017/3470320 PMC5440796 28572827

[B32] Yaribeygi H. Maleki M. Mohammadi M. T. Sathyapalan T. Jamialahmadi T. Sahebkar A. (2021). Crocin Improves Diabetes-Induced Oxidative Stress via Downregulating the Nox-4 in Myocardium of Diabetic Rats. Adv. Exp. Med. Biol. 1328, 275–285. 10.1007/978-3-030-73234-9_18 34981484

[B33] Zeng H. Zhu X. Tian Q. Yan Y. Zhang L. Yan M. (2020). *In Vivo* antitumor Effects of Carboxymethyl Chitosan-Conjugated Triptolide after Oral Administration. Drug. Deliv. 27, 848–854. 10.1080/10717544.2020.1770370 32508161 PMC8216443

[B34] Zhang L. Yan M. Chen K. Tian Q. Song J. Zhang Z. (2020). Novel Carboxylated Chitosan-Based Triptolide Conjugate for the Treatment of Rheumatoid Arthritis. Pharmaceutics 12 (3), 202. 10.3390/pharmaceutics12030202 PMC7150988 32110979

[B35] Zhang Y. Fei F. Zhen L. Zhu X. Wang J. Li S. (2017). Sensitive Analysis and Simultaneous Assessment of Pharmacokinetic Properties of Crocin and Crocetin after Oral Administration in Rats. J. Chromatogr. B. Anal. Technol. Biomed. Life. Sci. 1044-1045, 1–7. 10.1016/j.jchromb.2016.12.003 28056427

[B36] Zhang Y. Mao X. Li W. Chen W. Wang X. Ma Z. (2021). Tripterygium Wilfordii: An Inspiring Resource for Rheumatoid Arthritis Treatment. Med. Res. Rev. 41, 1337–1374. 10.1002/med.21762 33296090

[B37] Zhao J. Zhang F. Xiao X. Wu Z. Hu Q. Jiang Y. (2021). Tripterygium Hypoglaucum (Levl.) Hutch and its Main Bioactive Components: Recent Advances in Pharmacological Activity, Pharmacokinetics and Potential Toxicity. Front. Pharmacol. 12, 715359. 10.3389/fphar.2021.715359 34887747 PMC8650721

[B38] Zhao Z. Zhang Y. Gao D. Zhang Y. Han W. Xu X. (2022). Inhibition of Histone H3 Lysine-27 Demethylase Activity Relieves Rheumatoid Arthritis Symptoms via Repression of IL6 Transcription in Macrophages. Front. Immunol. 13, 818070. 10.3389/fimmu.2022.818070 35371061 PMC8965057

